# Mental Health Professionals’ Views on Artificial Intelligence as an Aide for Children Anticipating or Suffering the Loss of a Parent to Cancer: Helpful or Harmful?

**DOI:** 10.3390/children12060763

**Published:** 2025-06-12

**Authors:** Mary Rose Yockel, Marcelo M. Sleiman, Heather Doherty, Rachel Adams, Kimberly M. Davis, Hunter Groninger, Christina Sharkey, Matthew G. Biel, Muriel R. Statman, Kenneth P. Tercyak

**Affiliations:** 1 Lombardi Comprehensive Cancer Center, Georgetown University, Washington, DC 20057, USA; 2 Department of Psychology, The Catholic University of America, Washington, DC 20064, USA; 3 Section of Palliative Care, MedStar Georgetown University Hospital, Washington, DC 20007, USA; 4 Section of Palliative Care, MedStar Washington Hospital Center, Washington, DC 20010, USA; 5 Division of Child and Adolescent Psychiatry, MedStar Georgetown University Hospital, Washington, DC 20007, USA

**Keywords:** children’s bereavement, mental health interventions, parental cancer, artificial intelligence

## Abstract

**Purpose**: Assess mental health professionals’ attitudes regarding the timing and characteristics of therapeutic interventions for children whose parents have incurable cancer, and whether professionals would use artificial intelligence (AI) in these interventions. **Methods**: Professionals were surveyed about their therapeutic approaches to caring for children when parents have incurable cancer under different scenarios. Data from N = 294 (69% male, 72% white, 26% Latine, 56% rural or underserved communities) physicians, psychologists, social workers, hospital chaplains, community health workers, and others were analyzed. Attitudes surrounding the timing and characteristics of interventions across the parent’s cancer journey were compared, including how professionals believed interventions should attend to dimensions of the child or family, and if, how, and when AI technology could be introduced. **Results**: Across 10 dimensions of childhood, (1) the child’s premorbid exposure to traumatic events, (2) a surviving parent’s presence, and (3) the child’s age were important factors to consider when making mental health care decisions in this context. The professionals reported being more likely to introduce therapeutic resources as early as possible in the parent’s illness (i.e., upon diagnosis). Regarding the use of AI, 87% foresaw its role in supporting children’s mental health. While 93.2% agreed that a grieving child could be helped by interacting with an AI-generated likeness of the deceased parent, when AI’s use was contextualized in providing support for a child who lost a parent to cancer, only 49% believed AI was appropriate. The participants were conflicted over when AI could be first introduced, either upon a parent’s illness diagnosis (19.4%), during a parent’s treatment (19.0%), or as part of a parent’s hospice care (12.6%). None believed it to be appropriate following the loss of the parent to cancer. **Conclusions**: AI is increasingly present in children’s daily lives and quickly infiltrating health care with widely accessible mental health chatbots. Concerns about privacy, the accuracy of information, and the anthropomorphism of AI tools by children give professionals pause before introducing such technology. Proceeding with great caution is urged until more is known about the impact of AI on children’s mental health, grief, and psychological well-being in the context of parental cancer.

## 1. Background

### 1.1. Cancer and Parenting

Nearly 500,000 parents are diagnosed with cancer in the US annually, and about 25% of them have advanced disease without a cure. These parents often struggle to speak openly with their children about their illness [1]. Compared to children of parents who are healthy, children with seriously ill parents carry greater psychological burdens, and children who are unsupported after the death of a parent are more likely to experience traumatic stress and other psychiatric comorbidities [2,3,4,5].

The psychological preparation and support for children prior to a seriously ill parent’s death has a protective role in lessening complex childhood grief and problem behavior [3,6,7]. Providing mental health resources to families that aid in children’s coping is especially important given the US’s growing youth mental health crisis. In the US, the prevalence rates of anxiety and depression have risen in children and adolescents in recent years, while the accessibility of behavioral health providers has worsened due to burn out [8,9,10]. Many regions of the country also face mental health workforce shortages, with the majority of communities having limited numbers of child-serving mental health providers and few to no practicing child psychiatrists [9,11]. These rates are lowest in rural and high-poverty regions [9,12]. Additionally, the out-of-pocket costs of mental health services are often prohibitive for some families accessing care, as insurance coverage for mental health treatment is often inadequate [10].

### 1.2. Mental Health Applications

As a possible solution for the growing need for accessible mental health services, commercially created mental health apps have launched to assist with problems such as anxiety, depression, substance use, and insomnia. However, very few of these products have been clinically validated [13]. In addition to mental health apps, artificial intelligence (AI; e.g., computer systems that are able to perform tasks that normally require human intelligence) is currently reshaping the organization and delivery of mental health care due to its widespread digital access on smartphones and low cost. For example, many private companies are incorporating “chatbots”, or natural language processing systems that can interpret human speech and provide learned responses to written questions, into their therapeutic services [14]. The “behavioral health copilot” chatbot, offered through Woebot Health, interprets information submitted by users and delivers advice and self-care strategies based on a cognitive behavioral therapy model. This product is just one of many, and illustrates the ways AI contributes to mental health care in a public health context. A recent survey of behavioral health clinicians found that 77% believed chatbots were important in mental health care, and 80% anticipated prescribing AI-enhanced mental health apps in the future [15]. Research has also highlighted the possible benefits that the widely available Internet chatbot ChatGPT can provide psychotherapy patients—as it is free, easily accessible, and can offer adapted treatment plans [16]. Its advice generally takes a cognitive or mindfulness-based therapy approach, providing high-level summaries that are easily understandable to users.

However, many professionals have serious reservations about the automatization of mental health care delivery, as AI generators can misread clients’ tones and contextual factors and unintentionally provide misinformed advice or misguided information to users [17]. In terms of ChatGPT (with >800 million weekly active users worldwide), researchers have learned that the application is limited in the number of psychotherapeutic techniques it suggests, relying mainly on a one-dimensional approach and overlooking other forms of therapy. Additionally, ChatGPT’s responses tend to be basic and limited, lacking the ability to consider a holistic, patient-centered perspective—unlike in-person therapy, which offers a more comprehensive and personalized approach to client care [16]. More generally with AI, there are numerous ethical concerns about data-sharing and privacy, with the potential for users to over-disclose personal information to chatbots without the guarantees of confidentiality that are standard in traditional mental health services. While isolated individuals, or those who feel embarrassed to engage in mental health care, may be more apt to use such tools, there is risk that individuals could develop emotional connections and anthropomorphize these AI devices in ways that are unhealthy [14]. These factors become exponentially more concerning when those individuals are children, as they may be too young, inexperienced, or cognitively and socioemotionally immature to discern errors or assumptions made by AI tools. A study of how children understand and interact with voice-based computer assistants (such as Amazon’s Alexa) revealed that children overestimate the capabilities, reliability, and knowledge of AI smart systems [18]. Because of their human-like attributes, young children have been known to associate emotional, social, and moral states onto these devices, leading to the possibility of them forming social bonds with technologies that they falsely associate as human-to-human interactions.

### 1.3. Children’s Bereavement Counseling

In the context of anticipatory loss and grief counseling (including for parental cancer), AI has emerged as a tool for legacy-building and the remembrance of a deceased loved one by surviving family members. Specifically, AI and generative AI (i.e., technology that creates or recreates the voices, images, and conversations of those who are imaginary or were once real) [19,20] possess the capacity to simulate the writings, speech, or appearance of a person and create an entirely new content based on a library of information about that individual—including emails, text messages, and social media posts. Similar to AI chatbots, “griefbots” allow mourners to have two-way conversations with AI versions of the deceased through chat or video or audio messages to create what has been referred to as a “generative ghost” [21]. The psychological impact of communicating with these bots has yet to be tested empirically. While such tools may help with coping in the immediate aftermath of a loved one’s death, such as from cancer, there are a plethora of bioethical, psychological, and social concerns that interaction with such bots could predispose children to complex and long-term grief [22]. Researchers have expressed ethical concerns about these tools related to consent on behalf of the deceased who the generative AI is designed after, and the loved one who is engaging with the technology. Issues of postmortem privacy and the psychological well-being of all those who encounter these devices are of concern too [23].

Given the questions about the future of AI in children’s mental health care in anticipation of or suffering the loss of a parent to cancer, and the substantial impact of this experience on children’s long-term well-being, psychological research is well-poised to fill this gap with data regarding the attitudes of professionals who counsel these patients and their families. In this study, such professionals were surveyed to identify the most pertinent tools for promoting children’s adjustment during and following their parents’ cancer journey and to identify which aspects of childhood (e.g., age and developmental stage, family structure, culture, living environment) were most important to attend to when treating these families. Next, they were queried about when, during parents’ cancer journeys, they believed mental health support should first be introduced, and we compared and contrasted professionals’ practices under different scenarios—including the use of AI for intervention. This study aimed to identify critical elements of evidence-based programming to support the children of parents with advanced cancer within the context of generative AI becoming increasingly accessible to the public.

## 2. Methods

### 2.1. Design

This study was conducted in two phases and the ethical review and regulatory oversight was conducted and approved by the host university’s Institutional Review Board. In phase 1, key informant interviews examined when children’s mental health professionals believed psychological interventions should be initiated for children grieving the loss, or anticipating the imminent loss, of a parent to cancer. We also asked the interviewees to identify which characteristics of the child, parent, and family’s circumstances should be attended to when creating supportive interventions for this context. In phase 2, the findings from these interviews were used to develop and deploy a wide-reaching survey that queried providers about their beliefs regarding the most *important* or *ideal* factors to consider, and the resources to use when caring for bereaved children. The survey in phase 2 further asked about professionals’ preferences for practice when caring for bereaved children, which demographic factors of a child they believed most important to attend to (termed the “core dimesons of childhood”) when providing supportive care, and about their beliefs in the potential role of AI as a resource to aid children anticipating or suffering the loss of a parent to cancer.

### 2.2. Sample and Procedure

This study was designed to inform researchers and practitioners within the multidisciplinary specialty of supportive and palliative care in oncology, which includes children’s mental health professionals. Participants recruited for both phases 1 and 2 were reflective of the diversity seen on these health care teams, with an array of professionals from different disciplinary backgrounds, from internal medicine to psychiatry and psychology, nursing, social work, pastoral care, child life, and others.

Phase 1 included interviewing professionals identified by the study team using snowball sampling. All were familiar with the supportive and palliative care needs for children’s mental health in anticipating and experiencing the loss of a parent to cancer. At the conclusion of these interviews, each participant nominated others to provide complementary perspectives. These recommendations generated N = 15 additional individuals with relevant expertise who were interviewed in an iterative manner.

All interviews were transcribed verbatim to preserve the authenticity of the participants’ narratives. Using a content analysis approach, transcripts were systematically coded to identify recurring themes. An iterative coding process was applied to ensure thematic saturation, and inter-coder reliability was established through a collaborative review and discussion among members of the researcher team. This method allowed for an in-depth understanding of the participants’ responses. Based on the study’s conceptual model of socioecological determinants of children’s mental health and well-being [24], these professionals also suggested core principles of child development (e.g., biological, psychological, and social principles that influence children’s outcomes in response to trauma or loss). The narratives generated from this approach guided and informed the survey content for phase 2. This was, in part, necessitated by the lack of task-specific and well-established measures in the rapidly evolving field of mental health and AI. Thus, the study’s survey items were designed through an iterative process informed by both the literature on cancer bereavement and child development, and by direct consultation with phase 1’s children’s mental health professionals; a copy of the survey is available upon request to the corresponding author. These professionals helped the research team identify relevant domains such as children’s well-being, psychological aspects of bereavement, and quality of life in the context of parental loss to cancer and AI.

For phase 2, the survey participants were identified using professional email listservs in psychology, psychiatry, and palliative care and supplemented by posting on Meta’s social media platforms and incorporated with ethical behavioral research practices [25]. The online recruitment process provided brief information to potential study participants about the purpose of the project and instructed them to visit a study website. There, the participants were exposed to a description of the project. The potential participants completed an online screening form to confirm their eligibility based on their professional background, expertise, and clinical practice. Specifically, potential survey-takers were asked to verify that (1) they were a children’s mental health professional and (2) they had experience supporting children and families surrounding loss due to parental cancer or other long-term illness in a professional capacity (i.e., knowledge, education, training, licensing, or credentialing). Only participants who endorsed these items and passed the screening test were routed to the online survey.

The participants then provided informed consent and were surveyed online. The study was posted for approximately 48 h and the participants were offered a $10 incentive to an online retailer. Using these methods, the posting was viewed by N = 1046 social media accounts and received N = 357 responses. Due to the nature of our online study administration, it was not possible to calculate an overall survey response rate (i.e., the study team could not know the total number of eligible participants who viewed the posting and chose not to participate).

From the overall N = 357 of respondents, 66% accessed the survey through the online posting. Validity checks cross-referenced the respondents’ self- reported ages, occupations, and education levels, and also eliminated duplicate surveys or those deemed unreliable based on internal logic (N = 63). A total of N = 294 surveys were retained and included in the analyses (82%).

### 2.3. Background Variables

The participants self-reported their sociodemographic and professional practice characteristics (age, gender, race, ethnicity, educational background, employment type and setting).

### 2.4. Core Dimensions of Childhood

The participants were surveyed about 10 core dimensions of childhood derived from the interview phase, and these dimensions were ranked by importance (age, prior exposure to traumatic events, presence of co-parent in the household, race of the child, cultural or ethnic identification, gender, other adults/relatives important in the child’s life, religious traditions and spirituality, rural versus urban community setting, and socioeconomic status).

### 2.5. Children’s Supportive and Palliative Care When a Parent Has Cancer

Given the variability in the timing of the delivery of supportive and palliative care services to children and families anticipating or suffering the loss of a parent to cancer, we sought to compare and contrast how professionals currently practice on this topic (“current”), how they would prefer to practice on this topic (“preferred”), and how they would practice in a standardized clinical case scenario, incorporating both their current and preferred approaches (“standardized”). Guided by insights from the interviews, a standardized patient scenario was developed to constrain factors related to children’s coping and grief that may influence practice behavior (child age and gender, household structure, social support, and religiosity). The study did not explicitly identify the gender of the deceased parent or their child in order to minimize parent–child gender stereotyping. When probing for differences between “current”, “preferred”, and “standardized” practice, the respondents indicated when, in a parent’s cancer journey, they currently intervene with supportive services for the ill parent’s children, and we compared these responses to when they would intervene under ‘preferred’ and ‘standardized’ conditions.

### 2.6. Defining AI to Health Professionals

AI was described to professionals taking the survey as “automated smart systems that can perform tasks that typically require people”. Examples of AI given to professionals included “virtual assistants such as Apple’s Siri, Amazon’s Alexa, or Google’s Assistant”. Descriptions of AI’s current and potential capabilities included “technology [that can generate] and [recreate] the voices, images, and conversations of others…[who] may be both newly created fictional persons or the imitation of others who are or were once real”.

### 2.7. Measuring AI Attitudes

To measure professionals’ attitudes towards the use of AI in their daily lives and clinical settings, 3 Likert scale items were used (“1—strongly disagree”, “7—strongly agree”). The item inquired about common AI tools in their daily lives, if they could envision the potential of AI in supporting children’s mental health, and AI’s application to managing children’s grief following the loss of a parent to cancer. Higher scores indicated more favorable attitudes toward AI (Cronbach’s *α* = 0.78).

### 2.8. Exploring AI as an Intervention Tool for Children

The respondents were then asked to clarify how and when AI tools could be introduced, and who should have control over them. The participants indicated at which developmental stage (from infancy to young adulthood) they believed it appropriate to introduce an AI tool (“generative ghost”) to a child who has lost a parent to incurable cancer. If the professionals believed the tool should not be available to children of any age, they could select that option. Regarding when an AI legacy-building tool could be introduced to children during a parent’s cancer journey, the professionals indicated when they would introduce such a tool.

### 2.9. Management and Control of AI Intervention Tool

Finally, the professionals were asked who should be responsible for managing an AI legacy-building tool on behalf of a grieving child. The participants could select from one, several, or all of the following options: (1) the surviving caregiver; (2) other adults such as grandparents, aunts, uncles, or older siblings; (3) health professionals; (4) an outside third-party non-profit; (5) an outside third-party private company; (6) it (the AI tool) should not be available; (7) a write-in option.

### 2.10. Statistical Analysis

The statistical analyses were conducted in steps. First, descriptive statistics were used to describe the characteristics of the study participants, as well as each of the study variables. Next, a rank-order analysis was conducted using a Plackett–Luce model to examine the participants’ beliefs about core dimensions of childhood, followed by univariate and bivariate tests of clinical practice behaviors and attitudes. Finally, the study compared and contrasted the use of AI as a mental health tool across core dimensions and ill parents’ cancer journeys. As part of these steps, an examination of the potential demographic and professional education levels and practice or experience variables was considered; none were significantly associated with the outcomes of interest, suggesting heterogeneity in the responses that could not be attributed to these parameters.

## 3. Results

### 3.1. Respondents

The N = 294 participants who provided data (M age = 32.74, 69% male) included child life specialists (N = 80), psychologists (N = 77), psychiatric nurses (N = 45), mental health service workers (N = 31), other mental health providers (N = 25), social workers (N = 15), clergy members or pastoral counselors (N = 13), and psychiatrists (N = 8). Most participants in the sample had either practiced clinically for 1–5 years (43.9%) or 6–10 years (45.9%) (see Table 1).

### 3.2. Core Dimensions of Childhood

The participants’ responses to items regarding core dimensions of childhood are reported in Table 2. As shown in rank-order, premorbid exposure to traumatic events (i.e., adverse childhood experiences (ACEs)), the presence of a co-parent in the household, and children’s ages were considered to be the most important core dimensions when supporting a child through anticipatory loss or grief over parental death from cancer.

### 3.3. Timing of Interventions Relative to Ill Parents’ Cancer Journey

Current practice: Regarding the relative timing of the professionals’ first clinical encounters with children of patients with cancer, 54.1% engaged with families at the time of diagnosis, 20.0% during treatment, 7.5% upon the completion of treatment, 3.4% as part of hospice care, and 15.0% following the loss of the parent (see Table 3). As currently practiced, relatively more professionals engaged with families upon illness diagnosis or during treatment (74.2%) compared to following the loss of a parent to cancer (15.0%). When the intervention timing was parsed into two groups, with interventions occurring during or prior to a parent’s cancer treatment termed ‘early’ and those occurring after a parent’s completion of cancer therapy or later termed ‘late’, the professionals were over 8 times more likely to intervene ‘early’ in the disease trajectory than ‘late’ (odds ratio (OR) = 8.19, 95% confidence interval (CI) = 5.60, 12.11, *p* < 0.01).

Preferred practice: Regarding the professionals’ preferences about the timing of their first clinical encounters with children during their parents’ cancer illness, 34.0% preferred intervening at the time of the parent’s diagnosis, 21.1% during their treatment, 12.9% upon completion of parental treatment for cancer, 5.8% as part of hospice care, and 26.2% following the loss of the parent to cancer (see Table 3). As with current practice, the professionals preferred to intervene early in the parent’s cancer journey, although this preference was less pronounced. Specifically, when contrasting interventions that would occur during or prior to a parent’s cancer treatment (early), or after their completion of cancer therapy or later (late), the professionals were 1.5 times more likely to prefer intervening early than late (OR = 1.51, 95% CI = 1.07, 2.11, *p* = 0.016).

Standardized practice: When presented with the standardized case of a gender-neutral 12-year-old child who recently experienced the loss of their gender-neutral parent with a late-stage cancer, the professionals reported that the relative timing of when they would have first intervened (e.g., with print materials for the child’s family) would be upon the parent’s diagnosis (25.5%), during treatment (22.4%), upon completion of treatment (16.0%), as a part of hospice care (9.5%), or following the loss of the parent (26.2%) (see Table 3). When comparing early and late interventions, there were no observable differences for the standardized case (OR = 0.86, 95% CI = 0.61, 1.21, *p* = 0.41). However, in an effort to further explore whether more subtle differences may have existed between more discrete stages of the parents’ illness trajectory, the late interventions were divided into two stages—while the parent was still alive but curative treatment had ceased (middle) or after the parent’s death (late). The findings from this three-way comparison between “early” (upon diagnosis and during treatment), “middle”, and “late” interventions were highly significant. Specifically, the professionals were over 2.5 times more likely to intervene in the early stage than they were to intervene in the middle (OR = 2.7, 95% CI = 1.88, 3.88, *p* < 0.01) or late stage (OR = 2.60, 95% CI = 1.81, 3.74, *p* < 0.01). There were no observable differences in the timing of interventions between the middle and late stages (OR = 0.97, 95% CI = 0.66, 1.42, *p* = 0.97).

### 3.4. Artificial Intelligence

The professionals surveyed as part of this research reported regular use of common consumer AI platforms (e.g., Amazon’s Alexa), with 90.5% agreeing they use AI as a part of their daily routine (e.g., time, weather, music, news, and general information). The participants were in favor of the use of AI in their professional practice as well, with 86.8% agreeing they could envision the potential of AI in supporting children’s mental health (e.g., a chatbot that interactively delivers a standardized behavioral intervention). Additionally, 93.2% agreed that a grieving child could be helped by interacting with an AI-generated likeness of a parent following their death, provided that (prior to passing away) the parent gave their permission to do so and was instrumental in organizing the AI’s content, learning, and training library. Apart from the 5.1% of professionals who reported that this form of AI should not be available to grieving children, regardless of their developmental stage, many were in favor of incorporating AI resources with these children while they were either school-age (5–10 years old; 55.8%) or during adolescence (11–17 years old; 52%). The respondents were also open to incorporating AI with young adults (31.0%) and preschoolers (28.2%) as well, although very few believed it was appropriate to do so with very young children (0–2 years old; 8.2%).

### 3.5. Use of Artificial Intelligence During the Cancer Trajectory

In parallel to inquiries about traditional mental health resource introduction during the child’s grieving process during and following parental loss to cancer, the survey inquired whether the professionals believed that AI should be introduced as a tool in this context, and if so then when. Here, 49.0% reported that AI technology should not be available, while 51% were open to using it. Of those who endorsed its use, most believed that AI could be introduced upon a parent’s illness diagnosis (19.4%), followed by during a parent’s treatment (19.0%) and as part of a parent’s hospice care (12.6%). No participants responded that this technology should first be introduced upon the completion of treatment or following the loss of the parent to cancer. This is in contrast to the receptivity of professionals to using AI with bereaved children when it was not contextualized to the illness of cancer, where nearly all professionals reported that AI would be appropriate at a certain developmental stage (94.9%).

### 3.6. Management of AI Intervention

The professionals were asked who should be able to control artificial intelligence technology on behalf of a grieving child and were able to endorse multiple entities that they believed were suited for the role. In total, 62.6% of professionals surveyed agreed that a bereaved child’s surviving caregiver should be able to manage the technology, while 34% believed that other adult family members, such as grandparents or aunts and uncles, should be able to control the resource. When asked about entities outside the family system, 59.2% of the professionals endorsed management by health care providers, 30.6% by third-party non-profits, and 11.2% by third-party private companies (who may profit off the technology). In sum, while 6.5% of the respondents still held that AI technology should not be available as a resource for bereaved children, 93.6% still deemed it appropriate when managed by a specific person or group (e.g., a caregiver or health professional).

## 4. Discussion

This study sought to understand what children’s mental health care professionals believed to be the most important elements to consider when caring for bereaved children who lost their parents to cancer. Through phases 1 and 2, this project identified core dimensions of childhood that supportive and palliative care professionals consider important when making choices about therapeutic interventions for children anticipating or suffering the loss of a parent to cancer. It also explored the professionals’ views on the use of new and emerging mental health technologies, such as AI. According to those surveyed, premorbid exposure to traumatic events, the presence of a co-parent in the household, and the child’s age were considered to be essential to attend to in this context. The prior literature underscores the significance of accounting for and addressing ACEs in clinical treatment planning to promote children’s resilience and adjustment later in life [26]. Children’s bereavement research also emphasizes the importance of a child having another primary caregiver in their life before and following parental loss to help buffer them against further distress [1,27,28,29]. When creating psychosocial resources for this population, attending to a child’s developmental stage is essential because it may maximize their understanding of illness and death and minimize their fear and distress [29,30].

Regarding the relative timing of supportive interventions, the professionals consistently reported that intervening *at the time of a parent’s diagnosis* or while the parent was *undergoing treatment* was the optimal moment to introduce resources. The professionals largely intervened during these time points in their *current* practice and reported these stages as their *preferred* periods for intervention. However, when presented with a standardized patient in which a parent had already passed away from cancer, there was more heterogeneity in how professionals clinically approached the case. Specifically, they were more likely to intervene at stages in the illness journey *prior to* the end of the parent’s treatment for cancer compared to after treatment had concluded, upon their enrollment in hospice care, or after parental death. This is consistent with the research literature showing that when children are more informed and better prepared for their parent’s illness and prognosis, family communication and psychological outcomes improve [1,6,7].

In this study, we also learned that professionals anticipate using AI as part of their clinical practice over time. More than half (93%) agreed that a grieving child could be helped by interacting with an artificially generated likeness of a parent following their death. However, 49% believed that AI technology *should not* be available as an intervention for children who are grieving the loss of a parent to cancer. There are a few explanations as to why these differences in clinician endorsement may have varied so drastically between conditions. We propose it was due to the way each AI question was contextualized; the first asked mental health professionals to consider AI use in general bereavement, while the second query framed AI use in the aftermath of a cancer loss. These questions were intentionally designed and sequentially ordered to mirror how professionals make decisions about novel interventions to well-known problems, moving from the general to the specific. Something new (such as AI) may be broadly appealing because of its high capacity, low cost, ease of use, and claimed potential to change society—yet one must think more carefully about the scenarios, benefits, and harms it could evoke. The fact that children’s mental health care professionals were open to using AI is encouraging for patient benefit but perhaps even more encouraging is that they reflected with greater caution in a cancer context. Just as we saw in the data above, where the professionals’ opinions on the ideal timing of interventions during the cancer trajectory varied between the broader “preferred practice” query and the more specific “standardized” vignette, here too the opinions of the professionals changed as our framing changed from general bereavement to a specific and applied scenario.

Importantly, AI for bereavement use was endorsed by the professionals only if the ill parent gave permission, the children’s maturity level was taken into account, and if the artificially generated resource was being actively managed by a surviving caregiver or professional. These opinions align with current ethical concerns about AI technology in the field [18,23]. Specifically, scholars studying griefbots express serious concerns about the consent of the individual being immortalized (in the case of this study, the terminally ill parent) and those using the technology (i.e., the grieving child and their surviving caregiver) [23]. There are also extensive concerns about children forming overly close relationships with these generative AI technologies, as the research has found that when humanized as conversational agents, AI technologies such as Amazon’s Alexa are more likely to be seen by young children as trustworthy, human-like beings with feelings, personality, and agency [18]. Restricting how much children can interact with these devices and providing ample education about interactive AI’s capabilities is important for safeguarding children’s well-being when interacting with them. The professionals’ reports in this study underscore how caution and intentionality should be practiced when introducing AI technology such as a griefbot to a bereaved child who has lost a parent to an incurable illness, including advanced cancer. They also highlight that it may not be appropriate at all to introduce very young children to this technology, or children who have specifically lost a parent to cancer.

These findings may be controversial; AI has the ability to generate an infinite number of legacy-building elements in diverse formats, from an AI-created letter that mimics a deceased parent’s handwriting and vocabulary to enhanced photo images that show what a deceased parent may look like years into the future. As such technologies become more widely accessible, children’s mental health researchers must examine the psychological impacts that these products could have on bereavement.

With respect to the use of AI tools with cancer and its illness trajectory, no clear finding emerged; approximately equal proportions of professionals were and were not inclined to incorporate AI under this circumstance. Among those who did endorse its use, they felt that AI could be engaged earlier in the parent’s illness trajectory, which is consistent with findings about the timing of other mental health interventions for children with a parent with cancer [1,6].

## 5. Limitations

This study used a self-report survey distributed via listservs and through social media postings. While the postings were designed to target professionals working with children and families and a survey screener was employed to limit the study participants to children’s mental health professionals, there were finite means to verify these data. Strict internal validity checks were conducted to control for misrepresentation; data that were questionable and deemed unreliable were excluded. This study was also limited in the ways that AI was explained and queried about. The field of AI is rapidly evolving and being applied across a variety of sectors [16]. In this study, AI was defined for the professionals in terms of smart speaker systems (such as Amazon’s Alexa), chatbots, and griefbots. However, there are many more forms of AI that may be applicable to the field of palliative and bereavement care, which future studies should explore, along with non-verbal communications that AI tools cannot adequately capture and reflect at present. There are also concerns that gender biases exist within AI, which could influence how children interact with it. For these and other reasons, this study attempted to control for deceased parent-surviving child gender by making our standardized clinical case scenario of gender-neutral. Apps created by specialized mental health professionals (e.g., those trained in software engineering, with high cultural awareness and an inclusive mindset) could potentially address these and other limitations. Finally, open-ended items could be added to future surveys of professionals, and opinions of surviving parents could also be taken into consideration.

## 6. Conclusions

Numerous resources have been created within the fields of children’s mental health and palliative care to support families when a parent has incurable illness, such as an advanced cancer. Unfortunately, many families experience difficulty accessing these resources, either due to parents and caregivers being too overburdened by the parent’s illness to address their children’s emotional needs or difficulty within the health care system in identifying children needing support [3,29]. There is no standardized manner for how children’s mental health care professionals document whether a patient with an advanced cancer is raising children and is in a parenting role—often leading to children being overlooked for mental health services [31]. These service delivery challenges are compounded by the national shortage of such professionals in the US, making it difficult for children to receive timely care [32,33]. With insights from providers and parents, additional research should examine how AI can be leveraged to provide accessible counseling and support for children facing the loss of a parent to cancer, and those whose parent has passed away. Researchers and engineers in the private sector have already begun creating chatbots, such as Eterni.me and Replika, to preserve the legacy of deceased loved ones [20]. Thus, there is an urgent need to examine the ethics of using AI technologies with such children, including their developmental and emotional impacts. Toward that end, one must be mindful of the risks of gender bias and other prejudices that AI can introduce, including how children of different backgrounds might experience AI differently [34]. The recent research has highlighted that conversational AI systems, including those used by children for emotional support, may inadvertently reinforce gender stereotypes in socially significant interactions, raising apprehensions about the subtle shaping of a child’s emotional framework during vulnerable periods such as grieving the loss of a parent [34]. Additional research is also necessary to examine how effective generative AI is at providing psychotherapy and explore which dangers this technology may pose if it is someone’s only form of treatment. Until such time that these issues are clarified, professionals are cautioned against the use of AI for supporting the children of parents with cancer and to rely on more traditional methods of intervention.

## Figures and Tables

**Table 1:** Participant demographics (N = 294).

	M or N	SD or %
Age	34.7	8.7
Sex		
Male	203	69.0%
Female	91	31.0%
Total	294	100%
Race		
American Indian/Alaska Native	8	2.7%
Asian or Asian American	2	0.7%
Black or African American	69	23.5%
Native Hawaiian or Other Pacific Islander	0	0%
White or Caucasian	212	72.1%
Other	3	1.0%
Total	294	100%
Ethnicity		
Hispanic, Latin, or Spanish Origin	76	25.9%
Non-Hispanic, Latin, or Spanish Origin	218	74.1%
Total	294	100%
Education		
Less than high school	0	0%
High school diploma/GED	2	0.7%
Two-year degree/some college	22	7.5%
Bachelor’s degree	142	48.3%
Master’s, doctoral, or other professional degree beyond a bachelor’s	128	43.5%
Total	294	100%
Work in a rural or underserved region of the US		
Yes	165	56.1%
No	129	43.9%

**Table 2:** Rankings of childhood core dimensions.

	Relative Ranking	M Rankings *	SD **
Child’s exposure to prior traumatic events *In the research literature, this is commonly referred to as “adverse childhood events (ACES)”.*	1	3.5	2.8
Co-parent present in the household *Refers to the presence of another primary guardian that takes care of the child and lives permanently in the child’s household.*	2	4.2	2.8
Age of child *Refers to the chronological age of the child when intervention happens.*	3	4.3	3.0
Cultural and/or ethnic identification *Refers to the cultural and ethnic identity of the child, family, and surrounding community.*	4	5.0	2.7
Other adults/relatives (not a co-parent) present in the child’s life *Refers to the presence of adult relatives such as aunt/uncles/grandparents and non-relatives such as teachers/coaches during intervention.*	5	5.2	2.6
Gender of child *Refers to the non-biological gender identification of the child.*	6	5.3	2.7
Religious traditions and spirituality *Refers to any religious or spiritual traditions held by the child, family, or surrounding community.*	7	5.9	2.7
Rural vs. urban community *Refers to the surrounding area that the child is currently living in.*	8	5.9	2.9
Race of child *Refers to the racial identification of the child.*	9	6.2	2.8
Socioeconomic status *Refers to the socioeconomic status of the family and their surrounding community.*	10	6.2	3.2

* Mean ranking refers to the average ranking (1–10) each dimension received (1 being the most important, 10 being the least important). ** Standard deviation of the mean rankings.

**Table 3:** Timing of first clinical encounters by cancer trajectory across scenarios.

	Current		Preferred		Standardized	
Cancer Trajectory	n	%	n	%	n	%
Upon diagnosis	159	54.1 *	100	34.0 *	75	25.5 *
During treatment	59	20.1 *	62	21.1 *	66	22.4 *
Upon completion of treatment	22	7.5	38	12.9	47	16.0
As part of hospice care	10	3.4	17	5.8	28	9.5
Following the loss of the parent	44	15.0 *	77	26.2 *	77	26.2 *

Asterisks indicate significantly different comparisons across columns, * denotes *p* < 0.05. Percentage columns do not total to 100% due to participants’ ability to reply to multiple response options.

## Data Availability

The data presented in this study are not available due to privacy.
